# Combining Ivacaftor and Intensive Antibiotics Achieves Limited Clearance of Cystic Fibrosis Infections

**DOI:** 10.1128/mbio.03148-21

**Published:** 2021-12-14

**Authors:** Samantha L. Durfey, Sudhakar Pipavath, Anna Li, Anh T. Vo, Anina Ratjen, Suzanne Carter, Sarah J. Morgan, Matthew C. Radey, Brenda Grogan, Stephen J. Salipante, Michael J. Welsh, David A. Stoltz, Christopher H. Goss, Edward F. McKone, Pradeep K. Singh

**Affiliations:** a Department of Microbiology, University of Washington School of Medicine, Seattle, Washington, USA; b Department of Radiology, University of Washington School of Medicine, Seattle, Washington, USA; c St. Vincent’s University Hospital, Dublin, Ireland; d Department of Laboratory Medicine, University of Washington School of Medicine, Seattle, Washington, USA; e Departments of Internal Medicine and Molecular Physiology and Biophysics, Pappajohn Biomedical Institute, Roy J and Lucille A Carver College of Medicine, University of Iowa, Iowa City, Iowa, USA; f Howard Hughes Medical Institute, University of Iowa, Iowa City, Iowa, USA; g Department of Medicine, University of Washington School of Medicine, Seattle, Washington, USA; h Department of Pediatrics, University of Washington School of Medicine, Seattle, Washington, USA; University of Pittsburgh

**Keywords:** CFTR modulator, cystic fibrosis, *Pseudomonas aeruginosa*, *Staphylococcus aureus*, lung infection

## Abstract

Drugs called CFTR modulators improve the physiologic defect underlying cystic fibrosis (CF) and alleviate many disease manifestations. However, studies to date indicate that chronic lung infections that are responsible for most disease-related mortality generally persist. Here, we investigated whether combining the CFTR modulator ivacaftor with an intensive 3.5-month antibiotic course could clear chronic Pseudomonas aeruginosa or Staphylococcus aureus lung infections in subjects with *R117H-CFTR*, who are highly ivacaftor-responsive. Ivacaftor alone improved CFTR activity, and lung function and inflammation within 48 h, and reduced P. aeruginosa and S. aureus pathogen density by ∼10-fold within a week. Antibiotics produced an additional ∼10-fold reduction in pathogen density, but this reduction was transient in subjects who remained infected. Only 1/5 P. aeruginosa-infected and 1/7 S. aureus-infected subjects became persistently culture-negative after the combined treatment. Subjects appearing to clear infection did not have particularly favorable baseline lung function or inflammation, pathogen density or antibiotic susceptibility, or bronchiectasis scores on CT scans, but they did have remarkably low sweat chloride values before and after ivacaftor. All persistently P. aeruginosa-positive subjects remained infected by their pretreatment strain, whereas subjects persistently S. aureus-positive frequently lost and gained strains. This work suggests chronic CF infections may resist eradication despite marked and rapid modulator-induced improvements in lung infection and inflammation parameters and aggressive antibiotic treatment.

## INTRODUCTION

The genetic disease cystic fibrosis (CF) has been transformed by drugs that act on the basic CF defect, impaired anion conductance of the cystic fibrosis transmembrane conductance regulator (CFTR) channel (1). Studies of ivacaftor, the first highly effective drug of this kind (called CFTR modulators), showed that treatment improved subjects’ lung and digestive function and nutritional status and reduced pulmonary exacerbations (2).

A remarkable finding from studies of ivacaftor was that treatment had only modest effects on a cardinal manifestation of CF, chronic lung infections caused by Pseudomonas aeruginosa, Staphylococcus aureus, and other pathogens. For example, work using culture-based methods showed that ivacaftor produced rapid reductions in sputum P. aeruginosa density in chronically infected subjects and reduced lung inflammation (3). However, P. aeruginosa density rebounded after ∼1 year, and P. aeruginosa strains present pretreatment were found to persist for ∼6 years of follow-up (4). Epidemiological studies analyzing patient registry data (5–9) and studies using DNA-based methods (5, 10, 11) also indicate that chronic infections usually persist in modulator-treated patients. Persistent infection is likely to be detrimental, so strategies that eradicate infections in modulator-treated patients could markedly increase the health benefits from these drugs.

Antibiotics generally have only modest and transient effects in chronic CF infections (12–16). However, several findings raise the possibility that antibiotic efficacy could be increased following treatment with modulators. First, studies in CF pigs and humans indicate that improved CFTR function increases the activity of lung antimicrobials by raising airway pH (17, 18). Innate antimicrobials can be synergistic with antibiotics (19), so combining modulators and antibiotics could amplify the infection-reducing effects of each. Second, modulator-mediated reductions in bacterial density (as seen in reference 3) could increase antibiotic efficacy by the inoculum effect, a phenomenon wherein reduced bacterial density markedly increases antibiotic susceptibility (20, 21).

Third, modulators could reduce the stress-tolerant phenotype of infecting pathogens that causes resistance to killing. The tolerance phenotype is postulated to be due in part to nutrient and oxygen limitation in airway mucus (22–24), where pathogens mostly live (25, 26). Modulators reduce airway obstruction and increase mucociliary clearance (5), and these effects along with decreased pathogen density may diminish tolerance. Finally, reduced pathogen density could decrease intrastrain genetic diversity that is known to evolve during CF infections (27–30). This effect could increase antibiotic efficacy, if the abundance of drug-resistant variants were reduced as a consequence (31).

Together, these ideas led us to hypothesize that combining highly effective modulators with intensive antibiotic treatment might eradicate some P. aeruginosa and S. aureus infections in chronically infected people with CF. Here, we test this hypothesis, and we also present long-term follow up data on the effects of modulators on infection and inflammation in treated subjects.

## RESULTS

### Study design and subjects.

The rationale for combined treatment was the finding that chronic CF infections generally persist after ivacaftor. When this fact became known (3, 5), most ivacaftor-eligible subjects worldwide were already treated, and effective modulators for more common mutations did not exist. These points led us to seek out subjects with the rare ivacaftor-responsive *R117H-CFTR* mutation (32) who were not yet treated. Dublin, Ireland, has among the highest worldwide prevalence of *R117H-CFTR* subjects (allele frequency of 3% versus 1.04% in Europe and 0.7% worldwide) (33, 34), and the St. Vincent’s University Hospital in Dublin is a CF referral center with robust research capabilities. Thus, we were able to test combined treatment in a cohort of chronically infected subjects with at least one copy of *R117H-CFTR* at this center.

**FIG 1:**
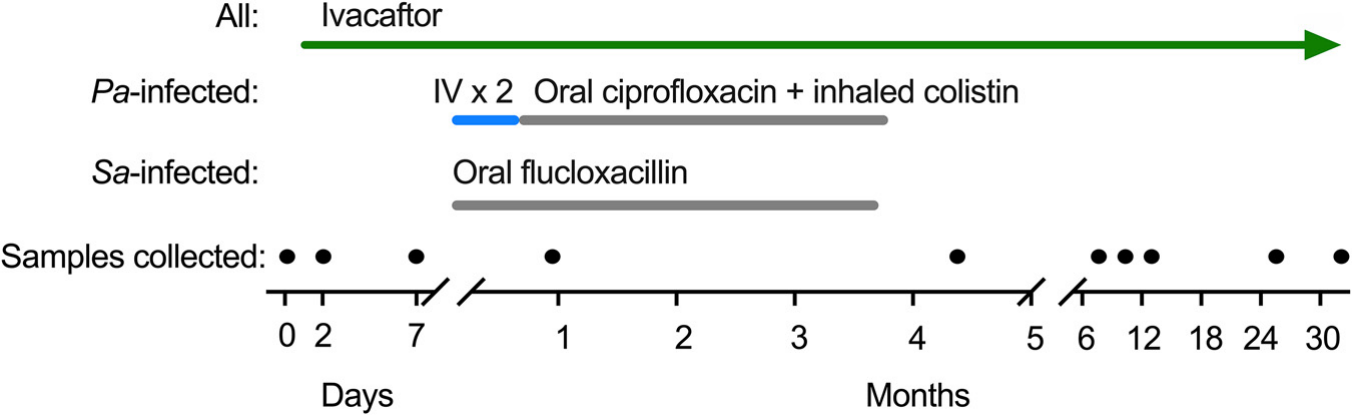
Study design. Subjects received ivacaftor for 1 week before initiating antibiotics. P. aeruginosa (*Pa*)-infected subjects received 2 weeks of 2 intravenous (IV) antibiotics simultaneously (including meropenem, tobramycin, colistin, or ceftazidime), followed by 3 months of oral ciprofloxacin and inhaled colistin simultaneously (see Table 1). S. aureus (*Sa*)-infected subjects received 3.5 months of oral flucloxacillin. We collected the first postantibiotic samples after a 1-month antibiotic-free washout period, and subjects were followed for 31 months in total; the filled circles indicate sample collection times.

Combined treatment could utilize antibiotics or modulators first or both simultaneously. We decided to use modulators alone for 1 week and then start antibiotics (Fig. 1) for three reasons. First, previous work showed that 1 week of ivacaftor reduced sputum P. aeruginosa density in subjects with a *CFTR* gating mutation (*G551D-CFTR*) by ∼10-fold (3). Reductions in this range are known to increase antibiotic activity via the inoculum effect (20, 21). Second, we thought there could be an advantage in starting ivacaftor and antibiotics in rapid succession to reduce time for bacterial adaptation. Third, the ivacaftor-alone period enabled us to repeat observations on modulators’ acute effects.

After ethics approval and informed consent, we prospectively studied 10 adults (aged 25 to 64) with CF and ≥1 *R117H-CFTR* allele. The average forced expiratory volume in 1 second percent predicted (FEV_1_pp) was 65%. Three subjects were chronically infected with P. aeruginosa, five were chronically infected with S. aureus, and two were infected with both. See Table 1 for additional subject characteristics and Table S1 for inclusion and exclusion criteria.

We used aggressive antibiotic regimes (Table 1). The 5 P. aeruginosa-infected subjects were treated for 14 days with 2 antipseudomonal antibiotics administered concurrently by IV, followed immediately by 3 months of oral ciprofloxacin and inhaled colistin administered together (Fig. 1). The 7 S. aureus-infected subjects were treated with 3.5 months of oral flucloxacillin, the drug of choice locally for S. aureus (Fig. 1).

**TABLE 1:** Subject characteristics and antibiotics prescribed^*a*^

Subject ID^*b*^	CFTR genotype: *R117H/*	Baseline FEV_1_ [L (% predicted)]	Age at entry (yr)	Gender	Pathogen(s) cultured at entry^*c*^	IV antibiotic	Oral antibiotic	Inhaled antibiotic
1	Δ*F508*	3.80 (108%)	45	Male	*Sa*	None	Flu	None
2	Δ*F508*	3.75 (96%)	40	Male	*Sa*	None	Flu	None
3	Δ*F508*	2.44 (87%)	41	Female	*Sa*	None	Flu	None
7	Δ*F508*	2.39 (69%)	52	Male	*Pa*, *Sa*	Cef, tob	Flu, cip	Col
8	*M156R*	1.37 (43%)	42	Female	*Pa*	Mer, col	Cip	Col
9	*M156R*	1.49 (35%)	40	Male	*Pa*, *Sa*	Cef, col	Flu, cip	Col
10	*2622 + 1G→A*	3.41 (75%)	25	Male	*Sa*	None	Flu	None
11	Δ*F508*	1.90 (62%)	46	Female	*Pa*	Cef, tob	Cip	Col
12	Δ*F508*	1.93 (57%)	53	Male	*Sa*	None	Flu	None
13	*3849 + 4A→G*	0.99 (57%)	64	Female	*Pa*	Mer, col	Cip	Col

a CFTR, cystic fibrosis transmembrane conductance regulator; FEV_1_, forced expiratory volume in 1 second; *Sa*, Staphylococcus aureus; *Pa*, Pseudomonas aeruginosa; flu, flucloxacillin; cip, ciprofloxacin; col, colistin; cef, ceftazidime; mer, meropenem; tob, tobramycin.

b There is no subject 6. Subjects 4 and 5 were excluded, as they were culture negative for all pathogens at trial entry, despite a history of culture positivity.

c Fungal colonization is presented in Table S2.

10.1128/mbio.03148-21.7 TABLE S1 Inclusion and exclusion criteria. Download Table S1, DOCX file, 0.01 MB. Copyright © 2021 Durfey et al. 2021 Durfey et al. https://creativecommons.org/licenses/by/4.0/ This content is distributed under the terms of the Creative Commons Attribution 4.0 International license.

10.1128/mbio.03148-21.8 TABLE S2 Fungi colonization at trial entry. Download Table S2, DOCX file, 0.06 MB. Copyright © 2021 Durfey et al. 2021 Durfey et al. https://creativecommons.org/licenses/by/4.0/ This content is distributed under the terms of the Creative Commons Attribution 4.0 International license.

### Ivacaftor improved sweat chloride, lung function, and inflammation within 48 hours.

**FIG 2:**
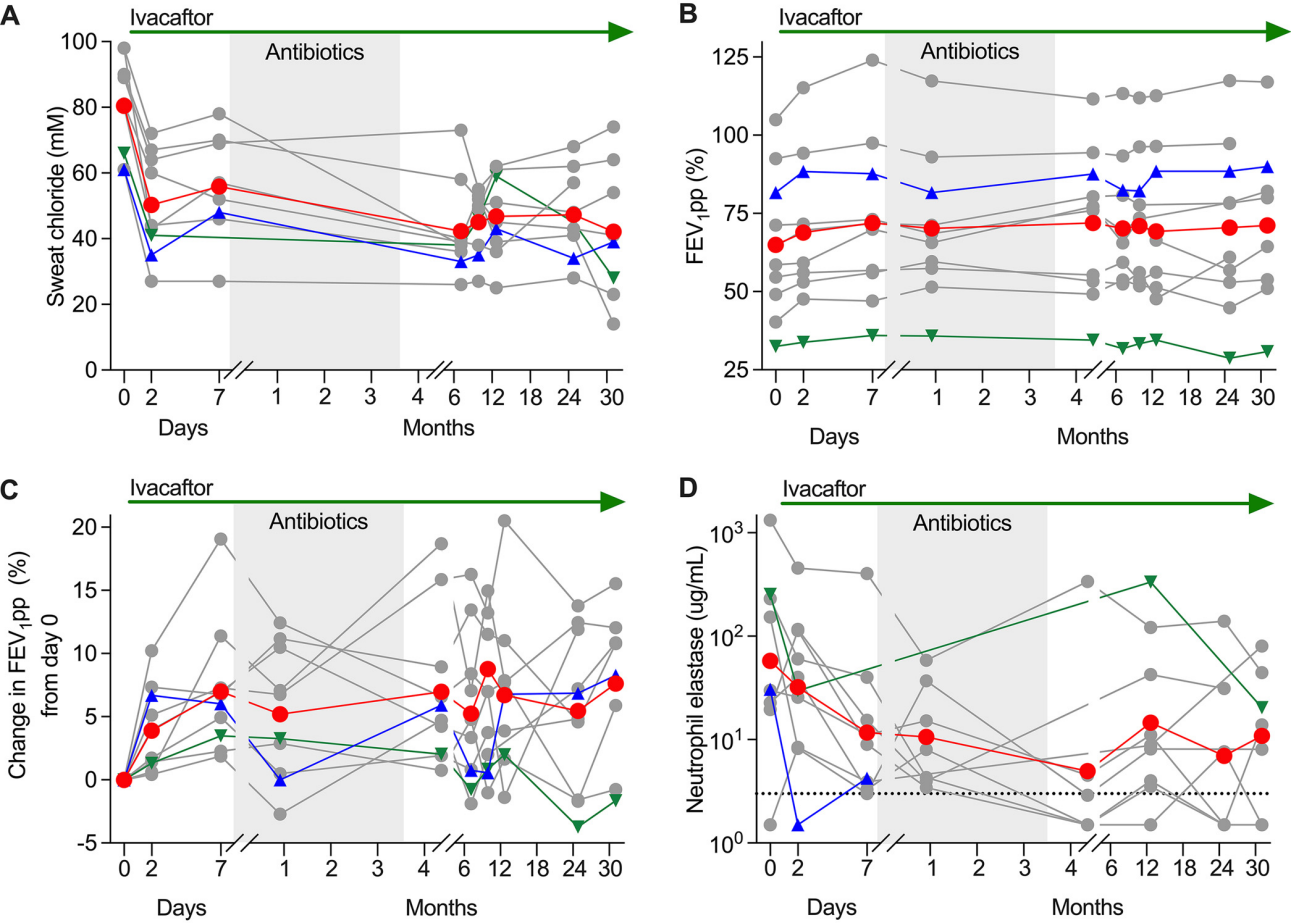
Ivacaftor rapidly improved CFTR and lung function, and lung inflammation. (A to D) Gray lines represent individuals, the red line represents the mean (A, B, C) or geometric mean (D), blue triangles represent subject 3, green triangles represent subject 9, ivacaftor treatment is indicated at the top, and the shaded region represents the on-antibiotic period. Subjects 3 and 9 are graphed differently because they became culture negative (see Results). Data with each line labeled by subject number are available in Fig. S2. (A) Sweat chloride. (B) Lung function as measured by forced expiratory volume in 1 second percent predicted (FEV_1_pp). (C) Change in FEV_1_pp from day 0. (D) Neutrophil elastase per mL sputum. The dashed line represents the limit of detection.

After 48 hours of ivacaftor-alone treatment, average sweat chloride decreased from 80.4 to 50.3 mM (95% confidence interval [CI], −41.7 to −18.5; *P* = 0.0002) (Table 2 reports means, multiple-comparison adjusted *P* values, and 95% CIs for all measurements) and did not decrease further by day 7 (Fig. 2A). Average FEV_1_pp improved from 65.0% to 68.9% after 48 hours (95% CI, 0.35 to 7.4; *P* = 0.03), with additional improvement to 72.0% at day 7 (95% CI, 1.7 to 12.3%; *P* = 0.01) (Fig. 2B and C). Sputum neutrophil elastase levels declined from 1.8 to 1.5 log_10_ μg/mL after 48 hours (95% CI, −1.1 to 0.6; *P* = 0.86) and then declined further to 1.1 log_10_ μg/mL at day 7 (95% CI, −1.4 to −0.003; *P* = 0.049) (Fig. 2D). Interleukin-1β (IL-1β), IL-8, and body mass index (BMI) did not change appreciably (Fig. S1). These responses are similar to those of *G551D-CFTR* subjects previously studied (3).

**TABLE 2:** Effects of ivacaftor on clinical parameters and inflammation^*a*^

Parameter	Mean	No.	Change from baseline		
			Mean	95% CI	*P* value (adj)
Sweat chloride (mM)					
Baseline	80.44	9			
2 days	50.33	9	−30.11	(−41.70 to −18.53)	0.0002
7 days	55.88	8	−24.57	(−35.99 to −13.14)	0.0008
2.5 yrs	42.13	8	−38.32	(−62.61 to −14.03)	0.0050
FEV_1_ (% predicted)					
Baseline	64.98	10			
2 days	68.87	10	3.88	(0.35 to 7.41)	0.03
7 days	71.98	10	7.00	(1.68 to 12.33)	0.01
2.5 yrs	71.14	8	6.16	(−1.34 to 13.66)	0.11
Neutrophil elastase (log_10_ ug/mL)					
Baseline	1.76	10			
2 days	1.51	10	−0.25	(−1.12 to 0.60)	0.86
7 days	1.07	9	−0.69	(−1.39 to −0.003)	0.049
2.5 yrs	1.04	7	−0.72	(−2.17 to 0.72)	0.38
IL-1β (log_10_ pg/mL)					
Baseline	3.69	10			
2 days	3.66	10	−0.03	(−0.92 to 0.85)	0.99
7 days	3.38	9	−0.31	(−1.21 to 0.60)	0.79
2.5 yrs	2.60	7	−1.09	(−2.63 to 0.44)	0.17
IL-8 (log_10_ pg/mL)					
Baseline	4.68	10			
2 days	4.70	10	0.02	(−0.71 to 0.75)	>0.99
7 days	4.50	9	−0.19	(−1.08 to 0.71)	0.97
2.5 yrs	4.16	7	−0.52	(−1.64 to 0.60)	0.48
MacConkey (log_10_ CFU/mL) (presumptive *Pa*)					
Baseline	6.62	5			
2 days	6.26	5	−0.37	(−2.50 to 1.77)	0.96
7 days	5.61	4	−1.02	(−3.59 to 1.56)	0.41
2.5 yrs	3.34	3	−3.28	(−8.42 to 1.85)	0.12
MSA (log_10_ CFU/mL) (presumptive *Sa*)					
Baseline	5.31	7			
2 days	4.98	7	−0.33	(−2.35 to 1.69)	0.99
7 days	4.19	6	−1.11	(−2.11 to −0.12)	0.03
2.5 yrs	3.49	4	−1.82	(−6.33 to 2.68)	0.40

a CI, confidence interval; adj, adjusted for multiple comparisons; FEV1, forced expiratory volume in 1 second; IL, interleukin; *Pa*, Pseudomonas aeruginosa; MSA, mannitol salt agar; *Sa*, Staphylococcus aureus.

10.1128/mbio.03148-21.1 FIG S1 Ivacaftor’s effects on lung inflammation and body mass index (BMI). (A to C) Gray lines represent individuals, the red line represents the geometric mean (A and B) or mean (C), blue triangles represent subject 3, green triangles represent subject 9, ivacaftor treatment is indicated at the top, and the shaded region represents the on-antibiotic period. (A) IL-1β per mL of sputum. (B) IL-8 per mL of sputum. (C) BMI. Download FIG S1, TIF file, 2.9 MB. Copyright © 2021 Durfey et al. 2021 Durfey et al. https://creativecommons.org/licenses/by/4.0/ This content is distributed under the terms of the Creative Commons Attribution 4.0 International license.

10.1128/mbio.03148-21.2 FIG S2 Individual subjects’ data for sweat chloride, lung function, and neutrophil elastase. Each line represents an individual, and each individual is identified with a unique shape and color combination; ivacaftor treatment is indicated at the top, and the shaded region represents the on-antibiotic period. (A) Sweat chloride. (B) Lung function as measured by forced expiratory volume in 1 second percent predicted (FEV_1_pp). (C) Change in FEV_1_pp from day 0. (D) Neutrophil elastase per mL sputum. The dashed line represents the limit of detection. Download FIG S2, TIF file, 1.8 MB. Copyright © 2021 Durfey et al. 2021 Durfey et al. https://creativecommons.org/licenses/by/4.0/ This content is distributed under the terms of the Creative Commons Attribution 4.0 International license.

### Ivacaftor rapidly reduced sputum P. aeruginosa and S. aureus density.

**FIG 3:**
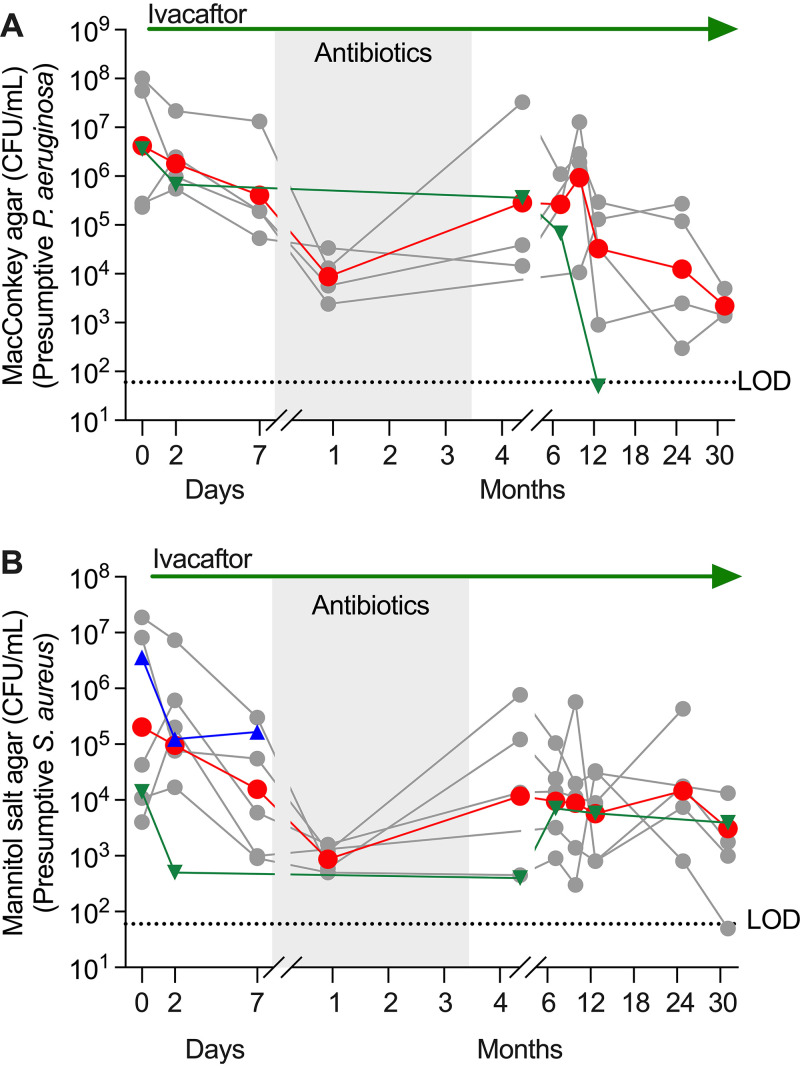
Ivacaftor rapidly improved sputum pathogen density. Gray lines represent individuals, the red line represents the geometric mean, blue triangles represent subject 3, green triangles represent subject 9, ivacaftor treatment is indicated at the top, the shaded region represents the on-antibiotic period, and the dotted line represents the limit of detection (LOD). Data with each line labeled by subject number are available in Fig. S3. (A) CFU per mL of sputum growing on MacConkey agar (presumptive Pseudomonas aeruginosa). (B) CFU per mL of sputum growing on mannitol salt agar (presumptive Staphylococcus aureus). See Fig. S4 for DNA-based measurements of pathogen density.

We measured P. aeruginosa and S. aureus density after the week of ivacaftor-alone treatment and found both decreased by ∼10-fold (P. aeruginosa mean change, −1.0 log_10_ CFU/mL [95% CI, −3.6 to 1.6; *P* = 0.41]; S. aureus mean change, −1.1 log_10_ CFU/ml [95% CI, −2.1 to −0.12; *P* = 0.03]) (Fig. 3). Changes were corroborated by PCR and sequencing analysis (Fig. S4) and similar to those of *G551D-CFTR* subjects previously studied (3). Importantly, an ∼10-fold reduction in bacterial density (as achieved here) can increase antibiotic efficacy by the inoculum effect (20, 21).

10.1128/mbio.03148-21.4 FIG S4 Calculated absolute abundance of pathogens corroborates CFU counts (see Fig. 3). Gray lines represent individuals, the red line represents the geometric mean, blue triangles represent subject 3, green triangles represent subject 9, ivacaftor treatment is indicated at the top, and the shaded region indicates the on-antibiotic period. Absolute abundance was calculated by multiplying the total bacterial abundance determined by 16S rRNA gene qPCR by the relative abundances of each amplicon sequence variant (ASV) determined by bacterial 16S rRNA gene sequencing. (A) Staphylococcus 16S copies per mL sputum. (B) Pseudomonas 16S copies per mL sputum. Download FIG S4, TIF file, 2.2 MB. Copyright © 2021 Durfey et al. 2021 Durfey et al. https://creativecommons.org/licenses/by/4.0/ This content is distributed under the terms of the Creative Commons Attribution 4.0 International license.

10.1128/mbio.03148-21.3 FIG S3 Individual subjects’ data for P. aeruginosa and S. aureus density. Each line represents an individual, and each individual is identified with a unique shape and color combination; ivacaftor treatment is indicated at the top, the shaded region represents the on-antibiotic period, and the dotted line represents the limit of detection (LOD). (A) CFU per mL of sputum growing on MacConkey agar (presumptive Pseudomonas aeruginosa [*Pa*]). (B) CFU per mL of sputum growing on mannitol salt agar (presumptive Staphylococcus aureus [*Sa*]). See Fig. S4 for DNA-based measurements of pathogen density. Download FIG S3, TIF file, 2.3 MB. Copyright © 2021 Durfey et al. 2021 Durfey et al. https://creativecommons.org/licenses/by/4.0/ This content is distributed under the terms of the Creative Commons Attribution 4.0 International license.

### Antibiotics further reduced pathogen density, but the decrease was transient.

P. aeruginosa and S. aureus density were reduced by an additional ∼10-fold after 3 weeks of antibiotics. P. aeruginosa decreased by 1.7 log_10_ CFU/mL (95% CI, −5.0 to 1.7; *P* = 0.23); S. aureus decreased by 1.3 log_10_ CFU/mL (95% CI, −4.0 to 1.5; *P* = 0.32) (Fig. 3 and Fig. S4).

We initially assayed for infection clearance 1 month after the 3.5-month antibiotic course had finished (and after 4.75 months of ivacaftor) to avoid residual antibiotic effects. Antibiotic-induced P. aeruginosa and S. aureus reductions were transient in most subjects, as pathogen density rebounded soon after antibiotics were stopped (Fig. 3) (see subsequent sections). However, one S. aureus-infected subject and one P. aeruginosa-infected subject became consistently culture-negative (discussed immediately below).

### One subject with chronic S. aureus infection became culture negative.

**FIG 4:**
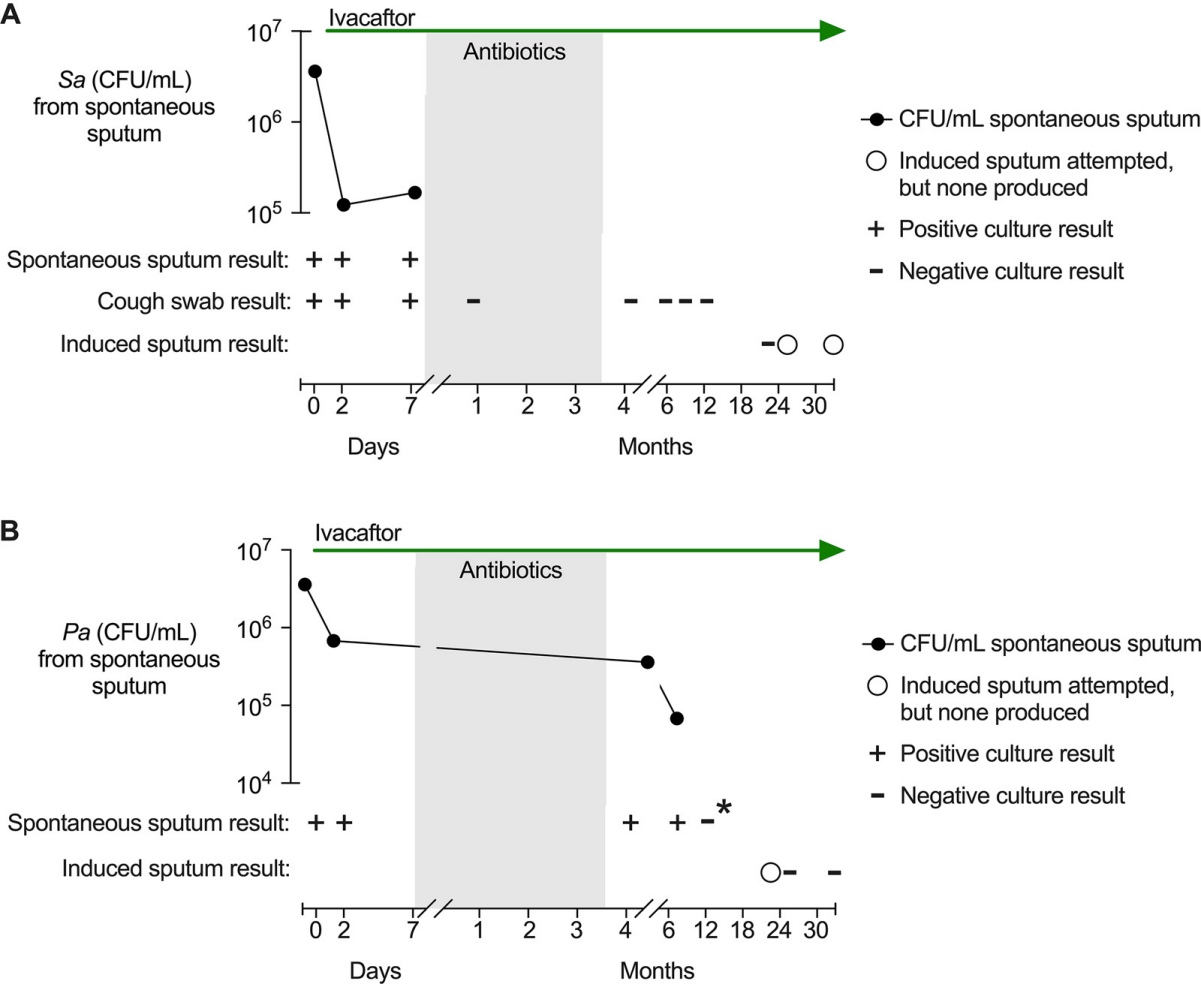
Ivacaftor and antibiotics reduced pathogen density below detectable levels in two subjects. Black lines represent CFU per mL in spontaneous sputum samples; pluses and minuses indicate culture results from each spontaneous sputum, induced sputum, or cough swab sample; and circles indicate times when induced sputum was attempted but unsuccessful. Ivacaftor is indicated at the top, and the shaded region represents the on-antibiotic period. (A) Staphylococcus aureus (*Sa*) culture results from subject 3. (B) Pseudomonas aeruginosa (*Pa*) culture results from subject 9. The asterisk (*) indicates that the culture result was confirmed with species-specific qPCR.

Subject 3 was infected with S. aureus for at least 15 years prior to the study (Table S3). Sputum cultures were S. aureus positive on days 2 and 7 while on ivacaftor alone (Fig. 4A). However, the subject became nonproductive for sputum after antibiotics (i.e., from day 7 to study end at month 31) (Fig. 4A).

10.1128/mbio.03148-21.9 TABLE S3 Infection histories of subjects 3 and 9. Download Table S3, DOCX file, 0.07 MB. Copyright © 2021 Durfey et al. 2021 Durfey et al. https://creativecommons.org/licenses/by/4.0/ This content is distributed under the terms of the Creative Commons Attribution 4.0 International license.

We anticipated that subjects could stop producing sputum, so we collected swabs from the posterior pharynx after forced coughing at every study visit. Control experiments indicated that swabs reliably reported S. aureus but not P. aeruginosa sputum culture positivity (Fig. S5). Swabs collected from subject 3 on days 0, 2, and 7 (when sputum was S. aureus positive) were S. aureus culture positive, whereas all swabs after antibiotics were negative (Fig. 4A). In addition, we made 3 attempts to collect induced sputum after inhalation of 7% saline. Only one attempt (month 23) yielded sputum, and it was S. aureus culture negative (Fig. 4A). These data suggest that the subject’s chronic S. aureus infection likely cleared.

10.1128/mbio.03148-21.5 FIG S5 Cough swab concordance with concurrently collected sputum samples. We tested the accuracy of cough swabs by culturing swabs that were collected concurrently with sputum. When the sputum sample was S. aureus culture positive (*n* = 7), 85% of cough swabs were S. aureus culture positive. However, when sputum samples were P. aeruginosa culture positive (*n* = 4), only 50% of swabs were P. aeruginosa culture positive. Download FIG S5, TIF file, 0.4 MB. Copyright © 2021 Durfey et al. 2021 Durfey et al. https://creativecommons.org/licenses/by/4.0/ This content is distributed under the terms of the Creative Commons Attribution 4.0 International license.

### One subject with P. aeruginosa infection became culture negative.

Subject 9 had a complicated infection history (Table S3). The subject became P. aeruginosa positive ∼1 year before treatment started and had P. aeruginosa-positive cultures on day 0 and day 2 during ivacaftor-alone treatment (Fig. 4B).

Sputum cultures at months 4 and 7 were also P. aeruginosa positive before the subject’s sputum became persistently P. aeruginosa negative after month 7 (Fig. 4B). The subject spontaneously produced sputum at month 13 that was P. aeruginosa culture and P. aeruginosa quantitative PCR (qPCR) negative (Fig. 4B) and then became nonproductive. We made 3 attempts to collect induced sputum (after inhalation of 7% saline). Two attempts yielded sputum (months 26 and 31) and were P. aeruginosa negative (Fig. 4B). Notably, subject 9’s sputum was consistently S. aureus positive (even after P. aeruginosa was no longer detected), and the subject suffered an exacerbation at month 26. Sputum before, during, and after the exacerbation was P. aeruginosa negative (Fig. 4B). Repeated negative cultures suggest that P. aeruginosa infection cleared.

### Subjects clearing infection did not harbor particularly sensitive isolates.

We investigated whether subjects becoming culture negative were outliers in some way, as this might suggest traits predisposing to infection clearance. We began by testing isolates’ baseline (i.e., before ivacaftor treatment) antibiotic sensitivities. P. aeruginosa isolates were tested against all agents used for IV, oral, and inhaled treatment (Table 1); S. aureus isolates were tested against flucloxacillin. Because pathogens can genetically diversify during infections, we tested the inhibitory concentration (IC) of 35 to 96 isolates from each subject and examined three parameters derived from the ICs of these populations of isolates. These included the median IC of the population from each subject, the IC defining the most resistant quartile of the population, and the IC of the most resistant isolate from the tested population, as we thought these could affect infection clearance.

**FIG 5:**
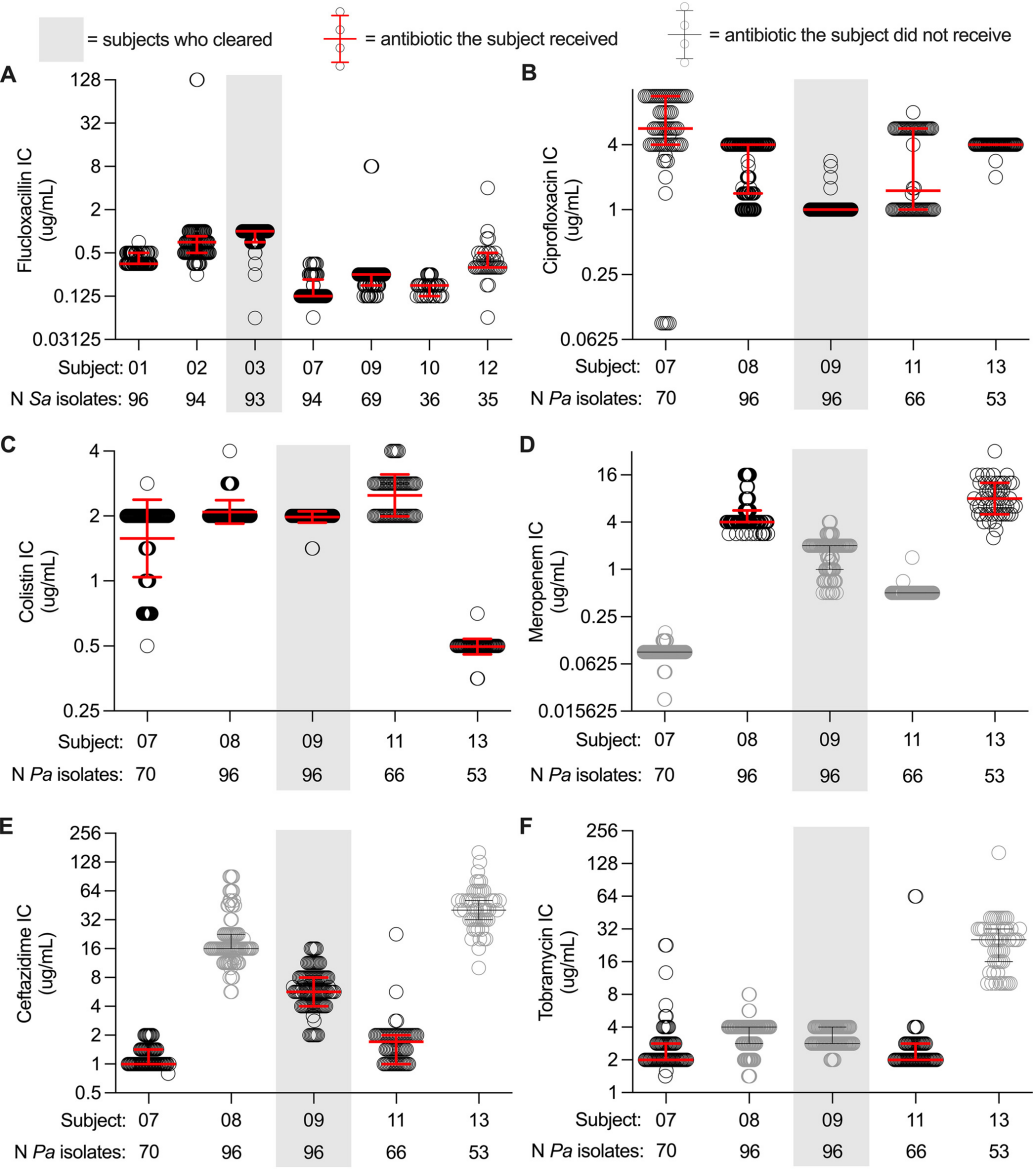
Subjects 3 and 9 do not harbor particularly sensitive bacteria. Each circle indicates the inhibitory concentration (IC) for each tested isolate (*n* = 35 to 96 per subject), and the error bars represent the median and interquartile range (IQR) calculated from the population of isolates. Results from antibiotics used to treat the respective subjects are reported using black circles with red error bars; results from antibiotics not used to treat the respective subjects are reported using gray circles with black error bars, and the shading indicates the subjects that cleared infection (3 and 9). (A) Staphylococcus aureus (*Sa*) flucloxacillin ICs. (B to F) Pseudomonas aeruginosa (*Pa*). (B) Ciprofloxacin ICs. (C) Colistin ICs. (D) Meropenem ICs. (E) Ceftazidime ICs. (F) Tobramycin ICs.

S. aureus isolates from subject 3 (who became S. aureus negative) were actually the most flucloxacillin resistant in the S. aureus cohort as measured by 2 of the 3 parameters (median and most resistant quartile) (Fig. 5A). P. aeruginosa isolates from subject 9 (who became culture negative for P. aeruginosa) were the most ciprofloxacin sensitive of the P. aeruginosa cohort by all three criteria (all P. aeruginosa-infected subjects received ciprofloxacin) but were not particularly sensitive to the IV or inhaled antibiotics this subject received (Table 1 and Fig. 5B to F). Moreover, persistently infected subjects also harbored unusually sensitive isolates (subject 13’s isolates were most sensitive to colistin, and subjects 7 and 11’s isolates were most sensitive to ceftazidime) (Fig. 5C and E). Thus, unusual antibiotic sensitivity (as measured *ex vivo* on cultured isolates) did not appear to account for infection clearance. However, we note that laboratory tests of antibiotic sensitivity may not reflect *in vivo* treatment efficacy.

### Subjects clearing infection had low baseline and ivacaftor-induced sweat chloride.

**FIG 6:**
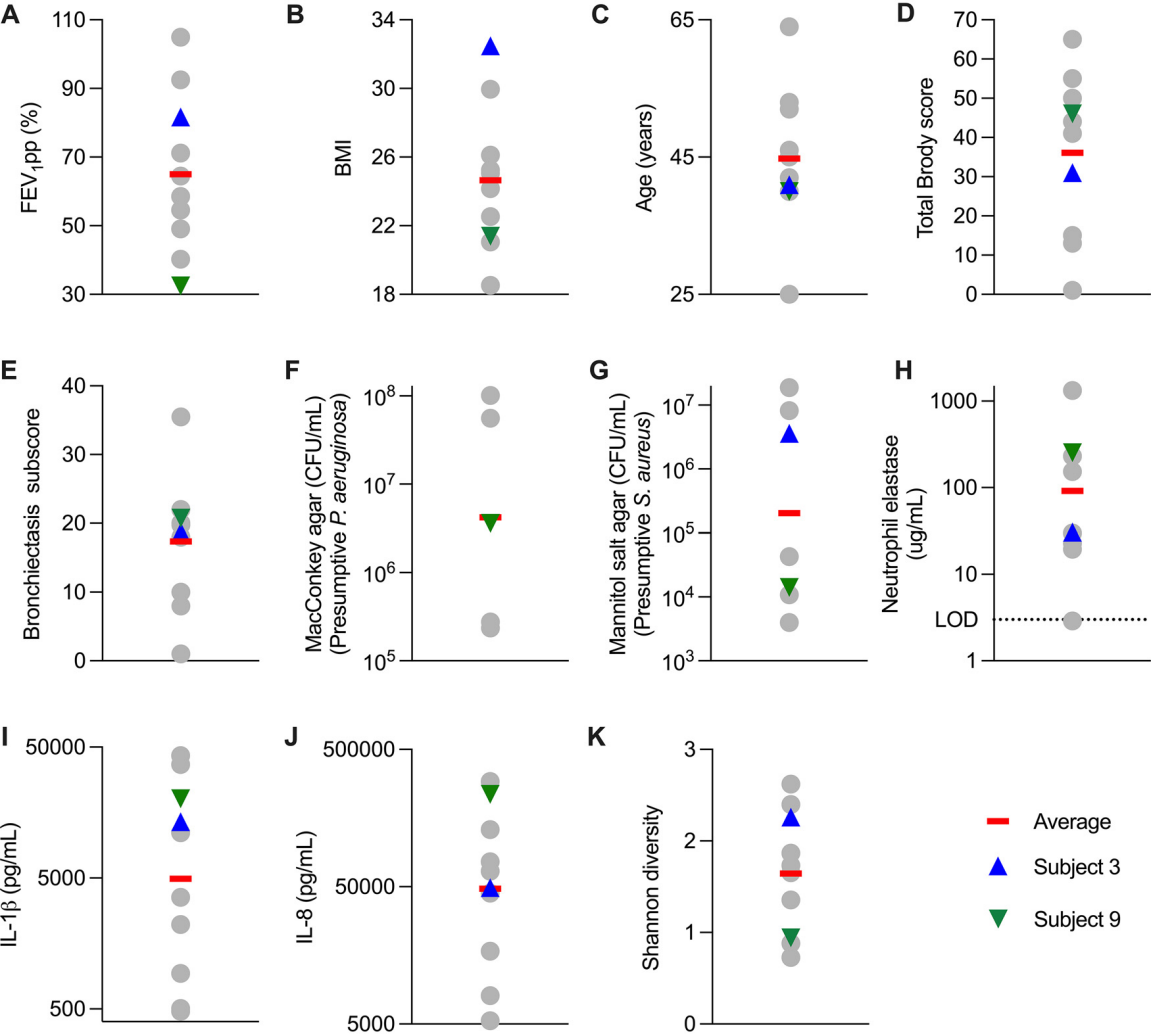
Subjects 3 and 9 clinical characteristics. Gray circles indicate individual subjects; the red line is the average (A to E, K, and L) or geometric mean (F to J), blue triangles indicate subject 3 (cleared S. aureus) and green triangles indicate subject 9 (cleared P. aeruginosa). All data are from baseline (day 0). (A) FEV_1_ percent predicted. (B) BMI. (C) Age. (D) Total Brody CT score. (E) Brody CT bronchiectasis subscore. (F) CFU per mL of sputum growing on MacConkey agar (presumptive Pseudomonas aeruginosa). (G) CFU per mL of sputum growing on mannitol salt agar (presumptive Staphylococcus aureus). (H) Neutrophil elastase per mL sputum. The dotted line indicates the limit of detection (LOD). (I) IL-1β per mL sputum. (J) IL-8 per ml sputum. (K) Shannon alpha diversity of sputum.

We also examined other characteristics of subjects who did and did not clear infection. Subjects clearing infection were not in the upper quartile of lung function or BMI. Nor were they in the lower quartile of age; lung injury on chest CT scans; baseline P. aeruginosa or S. aureus sputum densities; or sputum neutrophil elastase, IL-1β or IL-8 (Fig. 6A to J). Moreover, 16S rRNA sequencing indicated that subjects becoming culture negative had similar baseline sputum taxa relative abundance profiles (Fig. S6) and diversity (Fig. 6K) as persistently infected subjects.

10.1128/mbio.03148-21.6 FIG S6 Relative abundance of genera detected by 16S sequencing of sputum collected at baseline (day 0). Only the top 6 genera are shown. Subjects are divided into groups by culture result. Download FIG S6, TIF file, 1.4 MB. Copyright © 2021 Durfey et al. 2021 Durfey et al. https://creativecommons.org/licenses/by/4.0/ This content is distributed under the terms of the Creative Commons Attribution 4.0 International license.

**FIG 7:**
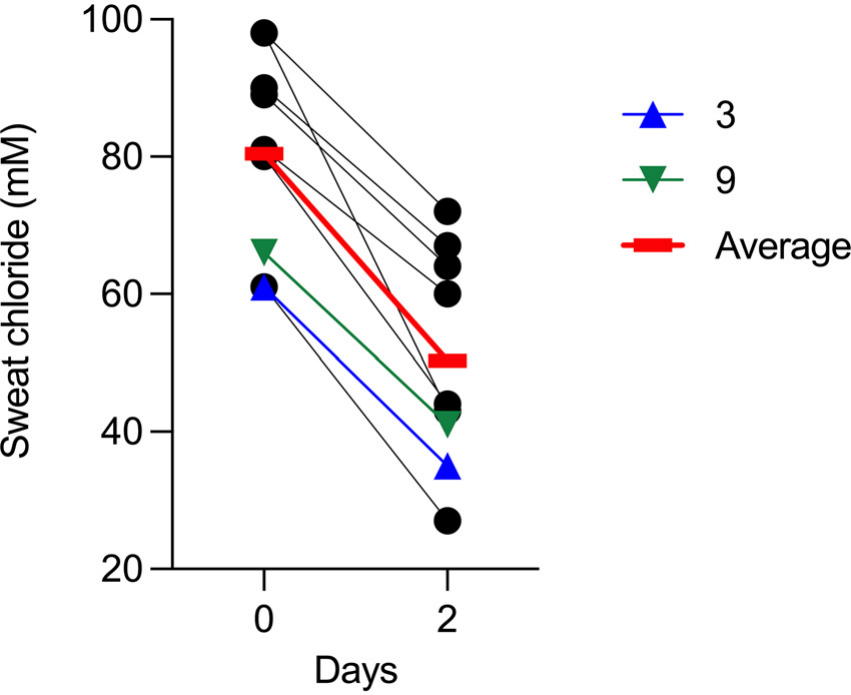
Subjects 3 and 9 had low baseline and ivacaftor-induced sweat chloride. Black lines represent individuals, the red line represents the mean, blue triangles represent subject 3 (cleared S. aureus), and green triangles represent subject 9 (cleared P. aeruginosa).

However, subjects 3 and 9 (who cleared S. aureus and P. aeruginosa, respectively) had among the lowest baseline sweat chloride values (61 and 66, versus an average of 80 mM for others) and achieved among the lowest values after ivacaftor (35 and 41 at 2 days, versus an average of 50 mM for others) (Fig. 7). These findings raise the possibility that infection clearance may depend in part on the amount of CFTR activity achieved (see discussion).

### Sweat chloride, lung function, and inflammation improved little after the first week.

Few data exist about modulators’ long-term effects, so we tracked key clinical parameters over 2.5 years (Table 2; Fig. 2). Notably, the acute sweat chloride and lung function improvements were sustained but did not increase. Average sweat chloride decreased by 30.1 mM from baseline to 48 hours, and 38.3 mM from baseline to 2.5 years (95% CI, −62.6 to −14.0; *P* = 0.005); average FEV_1_pp increased by 7.0% from baseline to 1 week, and 6.2% from baseline to 2.5 years (95% CI, −1.3 to 13.7; *P* = 0.11). Sputum neutrophil elastase followed a similar pattern with the first weeks’ improvements persisting over 2.5 years of follow-up; average neutrophil elastase decreased by 0.7 log_10_ μg/mL from baseline to 1 week, and 0.7 log_10_ μg/mL from baseline to 2.5 years (95% CI, −2.2 to 0.7; *P* = 0.38).

We also examined long-term trends in pathogen density (Fig. 3). Between the end of the antibiotic wash-out (month 4.75) and the study’s end (2.5 years), average P. aeruginosa density decreased by 2.1 log_10_ CFU/mL, but variability was high, so interpretation is difficult (95% CI, −9.3 to 5.1; *P* = 0.41). Average S. aureus density changed little during this time (95% CI, −7.9 to 6.7; *P* = 0.96).

### Subjects with persistent P. aeruginosa infection retained pretreatment strains, but S. aureus strains frequently switched.

While pathogen density rebounded ∼1 month after antibiotics, during combined treatment it was ∼100-fold lower than baseline (Fig. 3 and Fig. S4) and much lower than typical in CF. These marked reductions raised the possibility that pretreatment P. aeruginosa or S. aureus lineages were partially or completely replaced by new strains. We examined this possibility using PopMLST (35), a method that detects gain or loss of strains with high resolution by testing tens to hundreds of isolates per sample.

**FIG 8:**
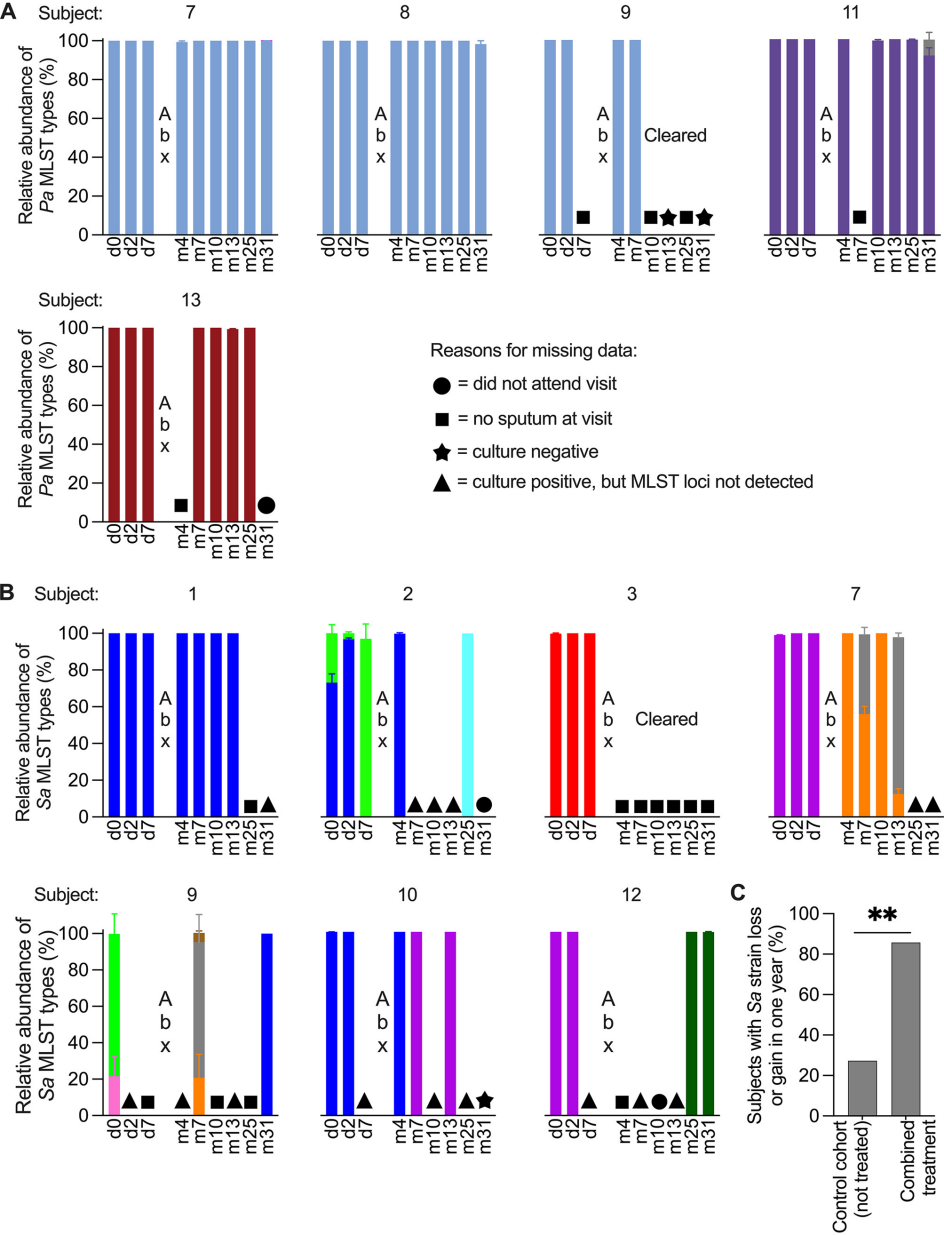
The relative abundance of MLST types recovered from subjects’ sputum. Each color represents a unique MLST type and is consistent across subjects; d, day; m, month; Abx, antibiotic period. MLST type abundances were determined using PopMLST (see reference 35) and represent the average relative abundance of alleles from 6 P. aeruginosa (*Pa*) or 7 S. aureus (*Sa*) MLST loci inferred to originate from a single strain. Where total relative abundance does not equal 100%, less than one-half the MLST loci exhibited a secondary allele, likely due to sequencing error. Symbols differentiate reasons for missing data (see key). (A) Relative abundance of P. aeruginosa MLST types. (B) Relative abundance of S. aureus MLST types. (C) Percentage of subjects who experienced at least one S. aureus strain gain or loss in 1 year for the control cohort (CF subjects not receiving combined ivacaftor + antibiotic treatment [*n* = 11]) and the combined treatment cohort (this clinical trial of ivacaftor + antibiotics [*n* = 7]) (**, *P* = 0.0022).

PopMLST performed on ∼95 P. aeruginosa isolates cultured from each time point showed that all cultured isolates from persistently infected subjects belonged to the single MLST type detected before treatment (Fig. 8A). These findings suggest that a single P. aeruginosa strain was dominant prior to treatment in all subjects and persisted through 2.5 years.

In contrast, S. aureus infections exhibited more strain diversity and switching. Baseline samples from 5/7 subjects contained 1 strain type, and 2/7 subjects were infected with 2 strains (Fig. 8B). Importantly, 6 of 7 (86%) of subjects either lost an existing S. aureus strain or acquired a new S. aureus strain during the first year of ivacaftor, with most strain switches becoming apparent at the first or second sputum sample obtained after antibiotics. For a comparison group, we examined banked samples from 11 additional S. aureus-infected CF subjects who were not treated with modulators and intensive antibiotics. Over a comparable time period (11.5 to 14 months), only 3 of 11 (27%) experienced S. aureus strain loss or gain (*P* = 0.002 compared to treatment group). These data and previous work showing that S. aureus strains generally persist for long time periods (36–38) suggest that modulators, antibiotics, or the combination compromised the stability of infecting S. aureus strains.

## DISCUSSION

Chronic lung infections are among the most consequential manifestations of CF, and studies to date indicate they generally persist after the basic CF defect is pharmacologically improved (3, 5–8, 10, 39). We tested an approach to improve outcomes by combining intensive antibiotics with modulators and studied the long-term effects of modulators in subjects with chronic infections.

We made four main findings. First, despite using intensive antibiotics in highly responsive subjects receiving highly effective modulators, only 1/5 P. aeruginosa-infected and 1/7 S. aureus-infected individuals cleared infection. Second, subjects clearing infection had particularly low baseline and post-ivacaftor sweat chloride levels, but we were not able to identify other distinguishing characteristics of these subjects. Third, subjects persistently infected with P. aeruginosa retained their pretreatment strains for 2.5 years of follow-up, but frequent strain switching was observed in S. aureus-infected subjects. Fourth, sweat chloride, lung function, and sputum neutrophil elastase markedly improved after 1 week of ivacaftor but showed minimal additional change over 2.5 years.

### Mechanisms that could explain infection persistence.

One explanation for persistent infection is that treated subjects could have significant residual lung host defense defects. Such defects could arise from at least three mechanisms. First, modulators produce incomplete restoration of CFTR function. This is the case in well-vascularized and uninflamed sweat glands (2); thus, lung cells in chronically infected subjects almost certainly have residual (and perhaps significant) CFTR dysfunction. Second, injury to lung epithelia and immune cells in regions with structural lung disease could restrict the beneficial effects of CFTR-targeting therapies to lung regions without such damage. Third, the continual presence of pathogens for years or decades could restrict improvements in innate antimicrobial activity due to immune tolerance mechanisms (40), even after CFTR function is restored.

A second explanation is that established chronic infections become autonomous or independent of CF host defense defects and would persist even if host defenses completely normalized. Lung defenses typically encounter a low density of inhaled or aspirated bacteria from environmental sources and clear these effectively. In contrast, modulator-corrected lung defenses must contend with high-density populations of pathogens that have genetically adapted and diversified over time (27–30, 41, 42). In addition, CF pathogens exhibit phenotypes that produce tolerance to killing, including low metabolic activity (43), an aggregated growth mode (25), and constitutive activity of stress responses (44, 45). These factors could make infection eradication challenging even if CFTR-dependent host defenses were normalized.

### P. aeruginosa strains persist, but S. aureus strains are transient.

It was interesting that P. aeruginosa lineages persisted, but S. aureus lineages were frequently gained and lost. The persistence of pretreatment P. aeruginosa strains could be due to specific pathogenic capabilities such as tolerance to antimicrobials (46, 47) or resistance to mechanical and phagocytic clearance (48, 49). Pathogen density may also contribute, as baseline average P. aeruginosa density was ∼10-fold higher that of S. aureus.

Our finding that almost all S. aureus-infected subjects receiving combined treatment gained or lost S. aureus strains during the first year of treatment was unexpected. Subjects studied in the premodulator era (36–38), and our control cohort not receiving combined treatment exhibited much lower rates of strain switching. This finding suggests that modulators, antibiotics, or the combination might destabilize existing S. aureus strains but not improve host defenses enough to prevent reinfection with new S. aureus strains. The ability of new S. aureus strains to infect after modulator treatment could be related to S. aureus’s ability to act as both a pathogen and commensal, as commensals can colonize sites in the absence of severe host defense defects. It is also possible that acquired S. aureus strains are inherently more capable in CFTR-corrected lungs than those that disappeared. If so, comparative genomics or phenotyping could identify differences.

### Persistently infected subjects exhibit most improvement in the first treatment week.

It was notable that sweat chloride, lung function, and neutrophil elastase improved in the first week with minimal additional improvements in the subsequent 2.5 years. P. aeruginosa and S. aureus density also showed 10-fold declines in the first week, with additional changes difficult to discern. These findings are consistent with previous observations (3) and suggest that benefits produced by improving lung defenses, inflammation, and physiology may not amplify each other over time to produce continual improvement. The mechanisms that limit improvement to the period immediately after drug initiation are unclear, but pathogen persistence and structural lung disease could be factors. Future work comparing lung function and inflammation responses in subjects with and without chronic infection would be informative.

### Study limitations.

Our study had several important limitations. First was the small study size. Several factors severely reduced subject availability. For example, we thought that the best test of an infection clearance regime would target chronically infected subjects with highly responsive *CFTR* mutations and that starting modulators and antibiotics in rapid succession might reduce time for bacterial adaptation. These considerations limited us to subjects with the rare *R117H-CFTR* mutation, as most subjects with *G551D-CFTR* were already receiving ivacaftor, and modulators for subjects with other *CFTR* mutation classes were not yet available. Furthermore, the study was burdensome and difficult to expand beyond a single center, as it required 3.5 months of IV, oral, and inhaled antibiotic treatment, immediate on-site sample processing, and close follow-up. Together, these factors restricted subject availability and may limit generalizability of the findings. The small study size also limited our ability to identify parameters (such as the extent of sweat chloride improvement) associated with infection clearance outcomes and to determine how infection clearance affects lung function (or other health outcomes). However, we do note that many study outcomes achieved statistical significance (*P ≤* 0.05 with multiple-comparison testing), including changes in sweat chloride, FEV_1_pp, neutrophil elastase, and S. aureus density.

Second, definitively proving that lung infections have been eradicated is challenging. Even the gold standard approach using bronchoscopy (which was not performed here) interrogates at most a few lung regions, and the sputum sampling used here may be less sensitive. However, repeated throat swabbing and spontaneous and induced sputum sampling in the two subjects that became culture negative support the conclusion that infection was cleared.

Third, the lack of a control group treated with ivacaftor but not antibiotics is a limitation. Prior studies indicate that chronic infections generally persist after modulator treatment and antibiotics when each are used alone (3, 5–11, 50). While this suggests that combined treatment could have caused infection clearance in the two cases, the absence of a control group makes this postulate tentative. Importantly, the lack of a control group does not compromise our central conclusion that chronic CF infections are difficult to clear even after aggressive use of antibiotics and modulators.

Finally, sampling issues somewhat limit the conclusions we can draw from the observation of S. aureus strain switching. For example, S. aureus’s ability to colonize upper airway tissues raises the possibility that the presence of some S. aureus strains in sputum arises from contamination from upper airway secretions, rather than originating from subjects’ lungs. In addition, no samples were banked from before treatment was initiated, so we were unable to determine how long pretreatment S. aureus strains were present before strain switching occurred.

This study highlights opportunities and challenges in treating chronic CF lung infections in the postmodulator era. It was encouraging that modulators produced rapid reductions in sputum pathogen density and inflammation markers and improved lung function. Our finding that infection clearance may be associated with low sweat chloride raises the possibility that infection clearance rates might be increased if CFTR function could be improved further. Studies of more effective modulators could test this idea. In addition, the high rate of strain S. aureus switching suggests that modulators may destabilize existing S. aureus strains, and this might be exploited therapeutically.

On the other hand, our finding that most subjects remained infected by P. aeruginosa and S. aureus despite highly effective modulators and intensive antibiotics points to challenges ahead. Future work will be needed to determine the extent to which persistent infection compromises health in modulator-treated subjects and to devise new strategies to eradicate chronic CF infections so that the full health benefits of CFTR modulators can be realized.

## MATERIALS AND METHODS

### Cohort characteristics.

This clinical trial of CFTR modulators plus antibiotics (EudraCT: 2016-001785-29) was performed at the National Referral Center for Adult Cystic Fibrosis at St. Vincent’s University Hospital in Dublin, Ireland. Subjects provided written informed consent prior to collection of samples. Subjects were people diagnosed with CF and heterozygous for the *CFTR-R117H* mutation. Individual subject characteristics are summarized in Table 1.

### Sweat chloride measurements.

Sweat was collected with the Macroduct collection system (Wescor, Logan, UT), and sweat chloride was measured using standard laboratory techniques.

### Spirometry.

Measurements were obtained using American Thoracic Society Standards (51). FEV_1pp_ is based on GLI 2012 values for adults (52).

### Computed tomography scans and scoring.

Images were obtained using 64-slice CT (Sensation 64; Siemens, Erlangen, Germany) with patients in the supine position. Inspiratory images were obtained in suspended deep inspiration with 1-mm slice thickness every 10 mm from the apices to the costophrenic angles. Expiratory images were obtained in full expiration at five levels—the top of the aortic arch, the carina, pulmonary veins, between level 3 and 5, and 2 cm above the diaphragm. Scanning parameters were 80 to 120 kilovoltage peak (kVp) and 50 to 120 milliamperes (mA), with images reconstructed using a high-spatial-frequency bone algorithm and a 512 × 512 matrix. Lung windows with a width of 1,500 hounsfield units and level of −700 hounsfield units were applied. Scans were reconstructed with filtered back projection and standard kernel, and they were deidentified prior to transfer to the University of Washington for analysis. For scoring, the 5 parameters of the Brody score (bronchiectasis, peri-bronchial wall thickening, mucous plugging, parenchymal damage, and air trapping) were measured in each of the 6 lobes of the lung (RUL, RML, RLL, LUL, Lingula, and LLL), and the sum of all scores was registered as the total Brody score (53, 54).

### Sputum processing for quantitation of bacterial concentration and inflammatory biomarkers, and culturing of P. aeruginosa and S. aureus.

Sputum specimens were homogenized with cold 0.1% dithiothreitol (DTT). One aliquot of homogenized sputum was serially diluted and plated on MacConkey agar (Difco) and mannitol salt agar (MSA; Difco) to quantify P. aeruginosa and S. aureus concentration, respectively. Up to 96 P. aeruginosa and S. aureus isolates were picked and made into freezer stocks.

### Quantification of sputum inflammatory biomarkers.

Homogenized sputum was processed according to the CF Therapeutics Development Network Coordinating Center standard operating procedure (3). IL-8 and IL-1β (Luminex multiplex bead; R&D Systems, Abingdon, Oxon, UK) and free neutrophil elastase activity (Spectrophotometric assay; Sigma Diagnostics, St. Louis, MO) were analyzed.

### Bacterial DNA extraction.

DNA was extracted from DTT-treated sputum using Qiagen’s DNeasy PowerSoil kit with modifications (3).

### Bacterial quantification.

Bacteria were quantified in duplicate with 16S rRNA qPCR primers and probe (“all bacteria” in reference 3) with Luna Universal Probe qPCR master mix (New England Biolabs [NEB]) with a CFX96 Touch real-time PCR detection system (Bio-Rad). Genomic DNA from P. aeruginosa PAO1 was used to generate a standard curve, which included a negative control (no template) and was run with every experiment.

### Bacterial 16S rRNA gene sequencing and analyses.

The V3-V5 variable region of the 16S rRNA gene was amplified with primers containing Illumina adapter sequences (Illumina 16S metagenomic sequencing library preparation). Demultiplexed sequencing reads were assigned to amplicon sequence variants (ASVs) using the DADA2 pipeline (55) (version 1.18). Shannon diversity was calculated in R (version 4.0.3) using the vegan package (https://github.com/vegandevs/vegan) and scripts adapted from Foster and Grünwald (56). ASVs were quantified by multiplying the total bacterial abundance determined by 16S rRNA gene qPCR by the relative abundances of each ASV determined by bacterial 16S rRNA gene sequencing, as in reference 57.

### Population-based multilocus sequence typing (PopMLST).

Detailed methods are reported in reference 35. The frozen P. aeruginosa or S. aureus isolates were inoculated into LB (for P. aeruginosa) or brain heart infusion (BHI) (for S. aureus) broth and grown overnight and then stamped onto LB or BHI plates using a 96-pin microplate replicator and grown overnight at 37°C. All isolates were scraped together using 1× phosphate-buffered saline (PBS), pelleted, and frozen for future DNA extraction with the Qiagen DNeasy PowerSoil kit, with modifications (3). See Table S4 for the number of isolates analyzed.

10.1128/mbio.03148-21.10 TABLE S4 Number of isolates tested for S. aureus and P. aeruginosa by PopMLST. Download Table S4, DOCX file, 0.07 MB. Copyright © 2021 Durfey et al. 2021 Durfey et al. https://creativecommons.org/licenses/by/4.0/ This content is distributed under the terms of the Creative Commons Attribution 4.0 International license.

We amplified the seven species-specific MLST loci using primers modified with the Illumina adapter sequence (35). We sequenced the amplicons on a 2 × 300-cycle cartridge (Illumina). Sequencing reads were processed using PopMLST software (https://github.com/marade/PopMLST). Briefly, reads were deconvolved based on their loci-specific primers, and then amplicon sequence variants (ASVs) were inferred using DADA2. To identify each allele, ASVs were queried against the PubMLST database (https://pubmlst.org/saureus/ and https://pubmlst.org/paeruginosa/) (58).

### Cough swab culture.

Due to limitations inherent in performing the study overseas, we froze the cough swabs in a glycerol-based transport medium (59). Thawed medium was serially diluted and plated onto MSA, Baird-Parker (Difco), MacConkey, and Pseudomonas isolation agar (PIA; Difco). P. aeruginosa positivity was defined as growth of at least one colony on MacConkey or PIA. S. aureus positivity was defined by phenotypes of growth on Baird-Parker and MSA, because both media also permit growth of Staphylococcus epidermidis.

### Antibiotic inhibitory concentration.

Briefly, the frozen isolates (day 0) were grown overnight in LB (P. aeruginosa) or BHI (S. aureus), stamped onto each plate with a 96-pin replicator, and grown at 37°C for 20 to 24 h. Each plate contained a different antibiotic concentration on a 2-log scale in LB. The inhibitory concentration (IC) of each isolate was recorded as the lowest concentration of antibiotic where the isolate did not grow; i.e., if an isolate grew at 2 μg/mL but not at 4 μg/mL, the IC was recorded as 4 μg/mL. P. aeruginosa isolates were tested on meropenem (obtained from clinic), tobramycin (RPI), ceftazidime (clinic), colistin (clinic), and ciprofloxacin (RPI). S. aureus isolates were tested on flucloxacillin (Sigma-Aldrich). All tests were done in duplicate, unless there was a >2-fold discrepancy.

### Control cohort for S. aureus strain switching.

Sputum samples were collected in accordance with University of Washington Institutional Review Board (protocol number 06-4469) from the adult CF clinic at University of Washington. Patients provided written informed consent prior to collection of samples. Samples were selected based on 2 criteria: (i) no history of CFTR modulator use and (ii) ≥2 banked S. aureus samples collected ≥1 year apart. Sputolysin-treated sputum was cultured on MSA. Populations were scraped from plates containing >100 colonies with LB. All cultures were stored at −80°C in 15% glycerol prior to analysis. About 100 μL of the glycerol-preserved sample was DNA extracted using the DNeasy PowersoilPro kit (Qiagen) for Qiacube (Qiagen). DNA was subjected to PopMLST as described above.

### Statistical analyses.

Repeated measure analysis of variance (assuming Gaussian distribution with a mixed model adjustment for missing data) was used to test FEV_1_pp, sweat chloride, culture, and inflammatory marker data. Where data were nonnormally distributed, they were first log-transformed. All *P* values are corrected for multiple comparisons, using Dunnett for comparisons to baseline and Šídák for comparisons between follow-up visits. A two-tailed *P* value of ≤0.05 was considered significant. To compare S. aureus strain switching, the control group was used as the expected and the combined treatment group was compared to this expected using a binomial test. Prism version 9.1 was used to perform all statistics and produce graphs.
