# Supplementary material for: Combining Ivacaftor and Intensive Antibiotics Achieves Limited Clearance of Cystic Fibrosis Infections
Source: mBio. 2021 Dec 14;12(6):e03148-21. doi: 10.1128/mbio.03148-21 (PMC8669489; doi:10.1128/mbio.03148-21)
Supplement: TABLE S1 [file mbio.03148-21-st001.docx]

**Table S1.** Inclusion and exclusion criteria.

| **Inclusion Criteria:**   - Adult, greater or equal to 18 years of age. - Documentation of a CF diagnosis with the *R117H-CFTR* mutation on at least 1 allele and any known or unknown second mutation other than *G551D-CFTR*. CF diagnosis is defined as evidenced by one or more clinical features consistent with the CF phenotype and one or more of the following criteria:   - 1. Sweat chloride ≥60 mEq/liter by quantitative pilocarpine iontophoresis test (QPIT)     2. Two well-characterized mutations in the cystic fibrosis transmembrane conductance regulator (CFTR) gene     3. Abnormal nasal potential difference (change in NPD in response to a low chloride solution and isoproteronol of less than -5 mV) - Prior chronic *Pa* or *Sa* in the sputum as defined by >50% of prior sputum cultures with each organism in the year prior to enrollment. Patients need a minimum of 2 prior cultures in the year. - Able to expectorate sputum. - Be clinically stable at the time of initiation of ivacaftor (day 0). - Be off chronic inhaled or oral antibiotics (other than azithromycin) for 2 weeks prior to free enrollment - Patients must be able to tolerate planned antibiotic regimen (anti-staphylococcal and anti-*Pseudomonas aeruginosa* regimens). - Written informed consent and assent if indicated obtained from subject or subject’s legal representative, able to communicate with the investigator and comply with the requirements of the protocol - If female and of childbearing potential, must have a negative pregnancy test on Day 1(Start of Treatment) prior to receiving study drug. - If female and of childbearing potential, is willing to use adequate contraception for the duration of the study and for 1 month following the study, as determined by the investigator. - If male and able to father a child, is willing to use adequate contraception for the duration of the study and for 1 month following the study, as determined by the investigator. |
| --- |
| **Exclusion Criteria:**   - Participation in the VX-770-105, VX-770-106, VX-770-108, VX-770-109, VX-770- 110, VX-770-111, VX-770-112, or VX-770-113 study, VX-770 Extended Access Program, VX-661-108, or use of ivacaftor within 6 months prior to day 0. - Any upper or lower respiratory symptoms requiring treatment with oral, inhaled or IV antibiotics within the 2 weeks prior to day 0. - History of solid organ transplantation. - Presence of a condition or abnormality that in the opinion of the investigator would compromise the safety of the patient or the quality of the data. - History of massive hemoptysis (>240 mL) within 72 hours of day 7. - Inability to produce sputum. - Females who have a positive pregnancy test at day 2, are lactating, or are not practicing (or willing to practice) a medically acceptable form of contraception (acceptable forms of contraception: hormonal birth control, intrauterine device, barrier method plus a spermicidal agent or abstinence) from day 2 through month 10 unless surgically sterilized or postmenopausal. |
