# Supplementary figures and images for: Combining Ivacaftor and Intensive Antibiotics Achieves Limited Clearance of Cystic Fibrosis Infections
Source: mBio. 2021 Dec 14;12(6):e03148-21. doi: 10.1128/mbio.03148-21 (PMC8669489; doi:10.1128/mbio.03148-21)

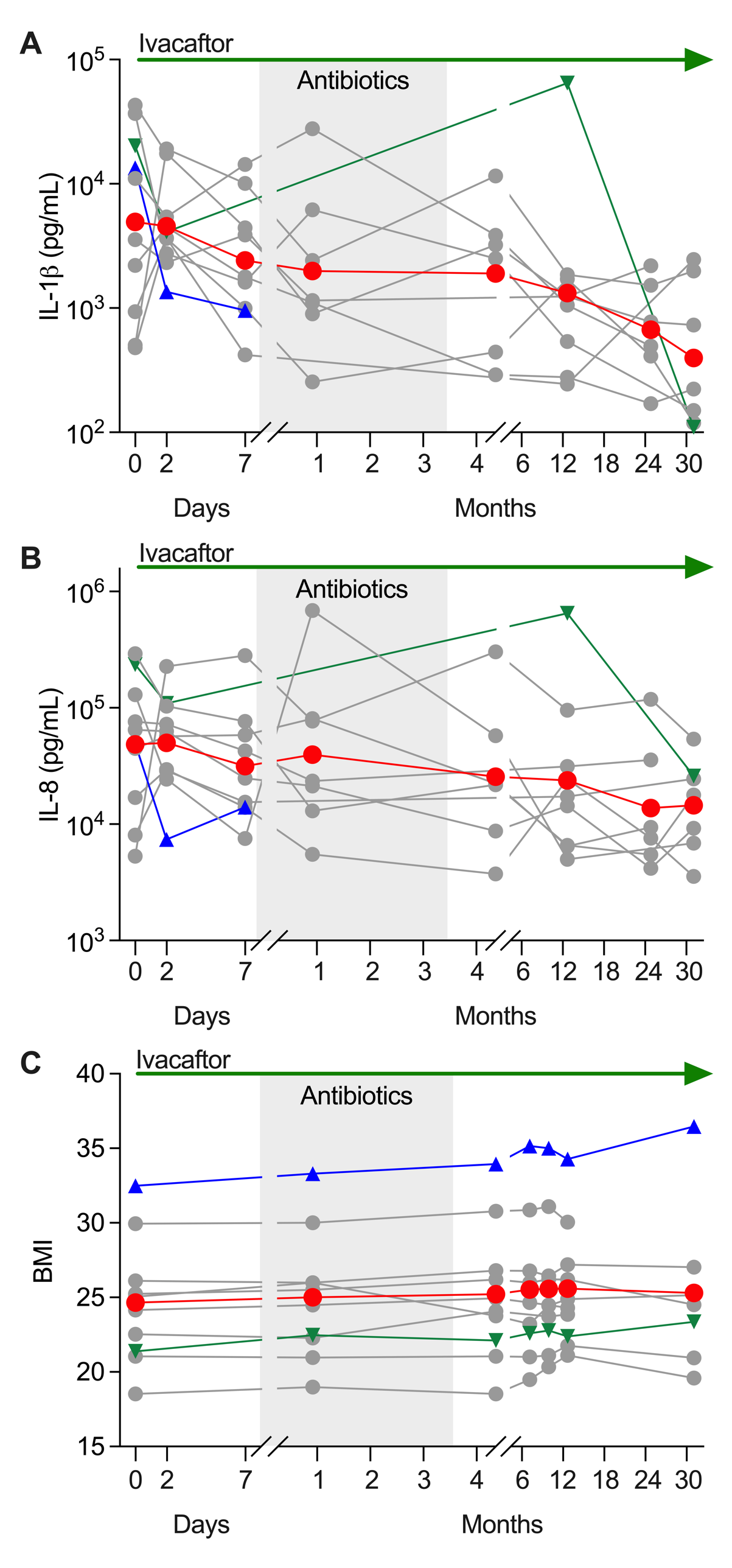

Supplement: FIG S1 [file mbio.03148-21-sf001.tif]

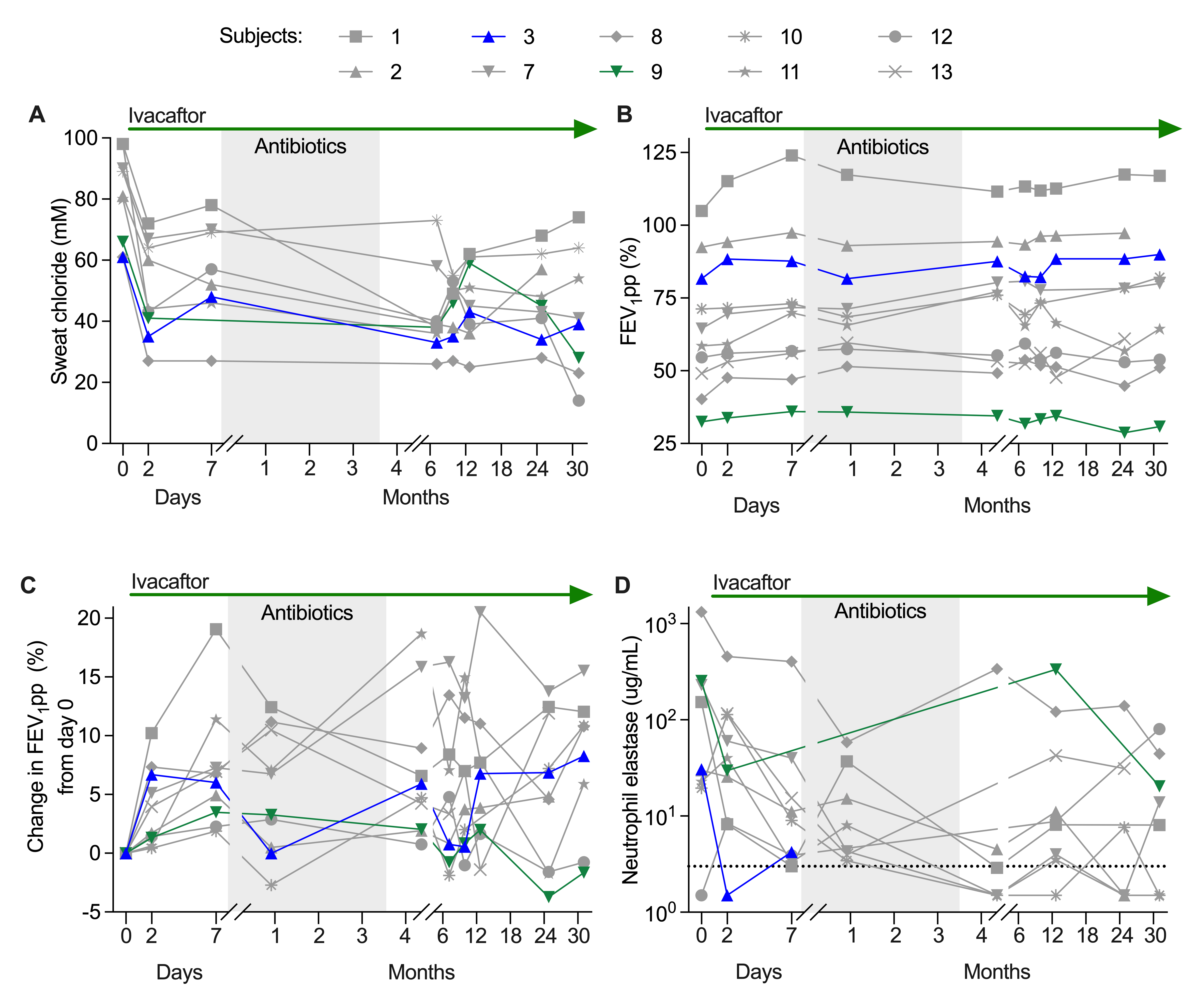

Supplement: FIG S2 [file mbio.03148-21-sf002.tif]

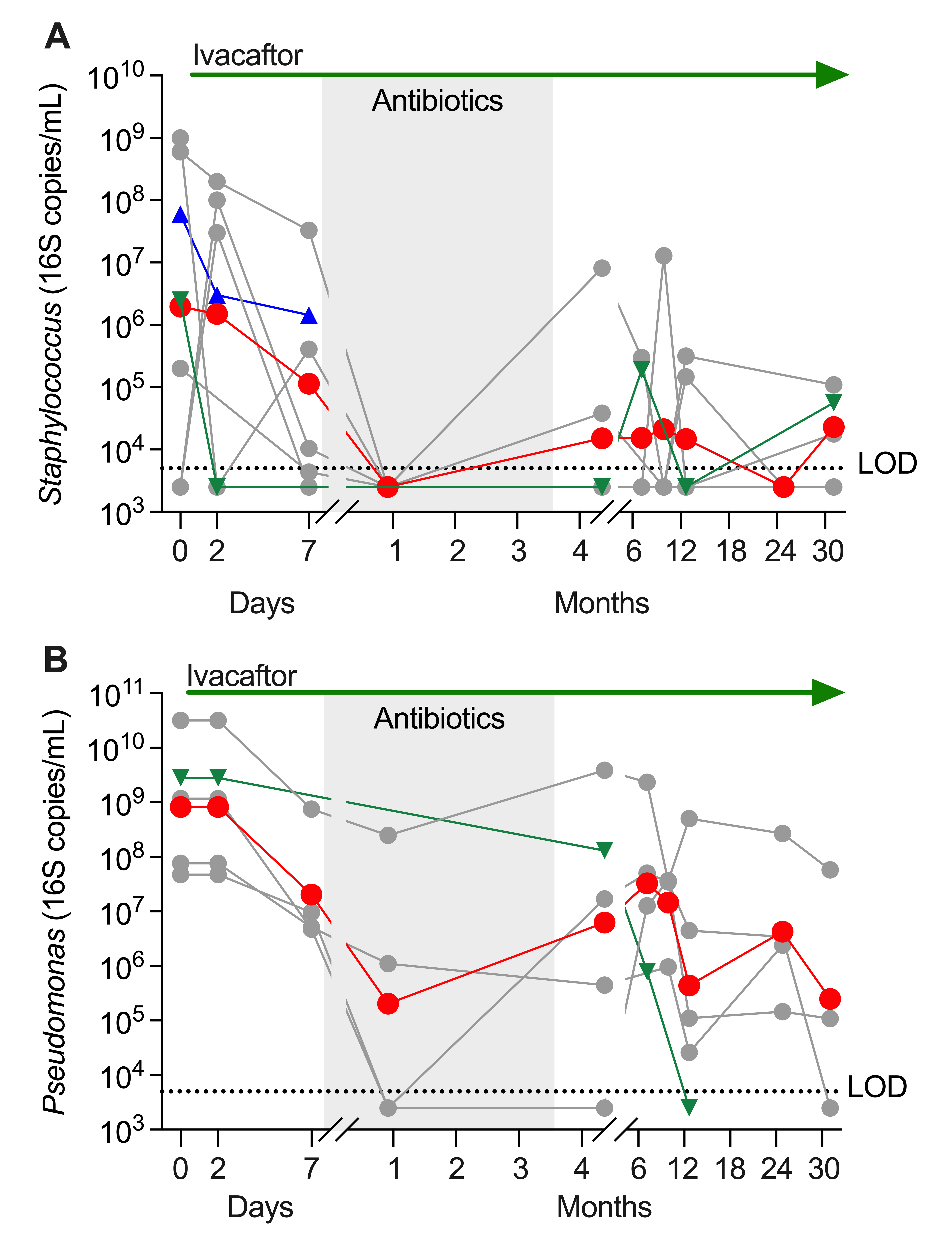

Supplement: FIG S4 [file mbio.03148-21-sf004.tif]

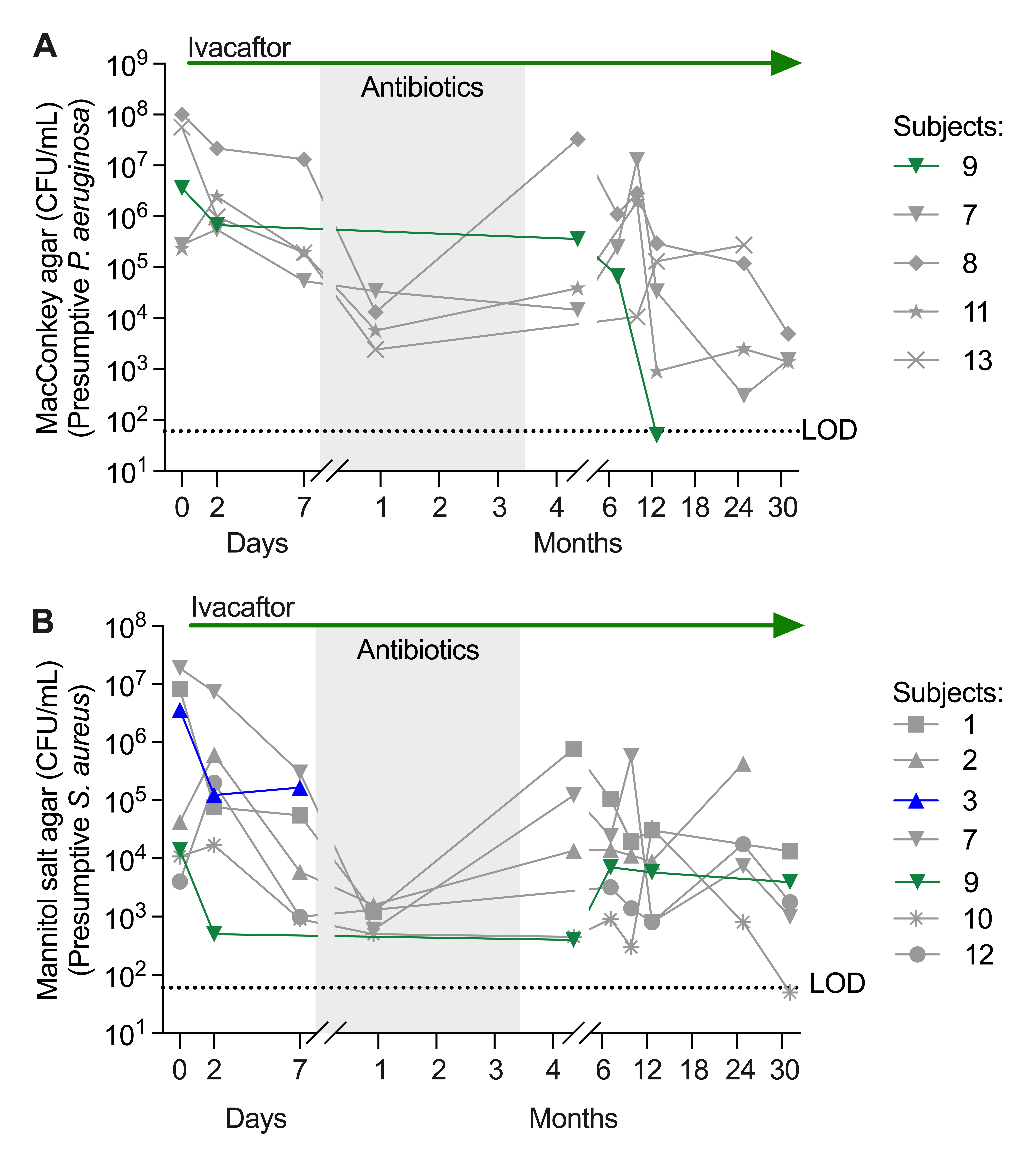

Supplement: FIG S3 [file mbio.03148-21-sf003.tif]

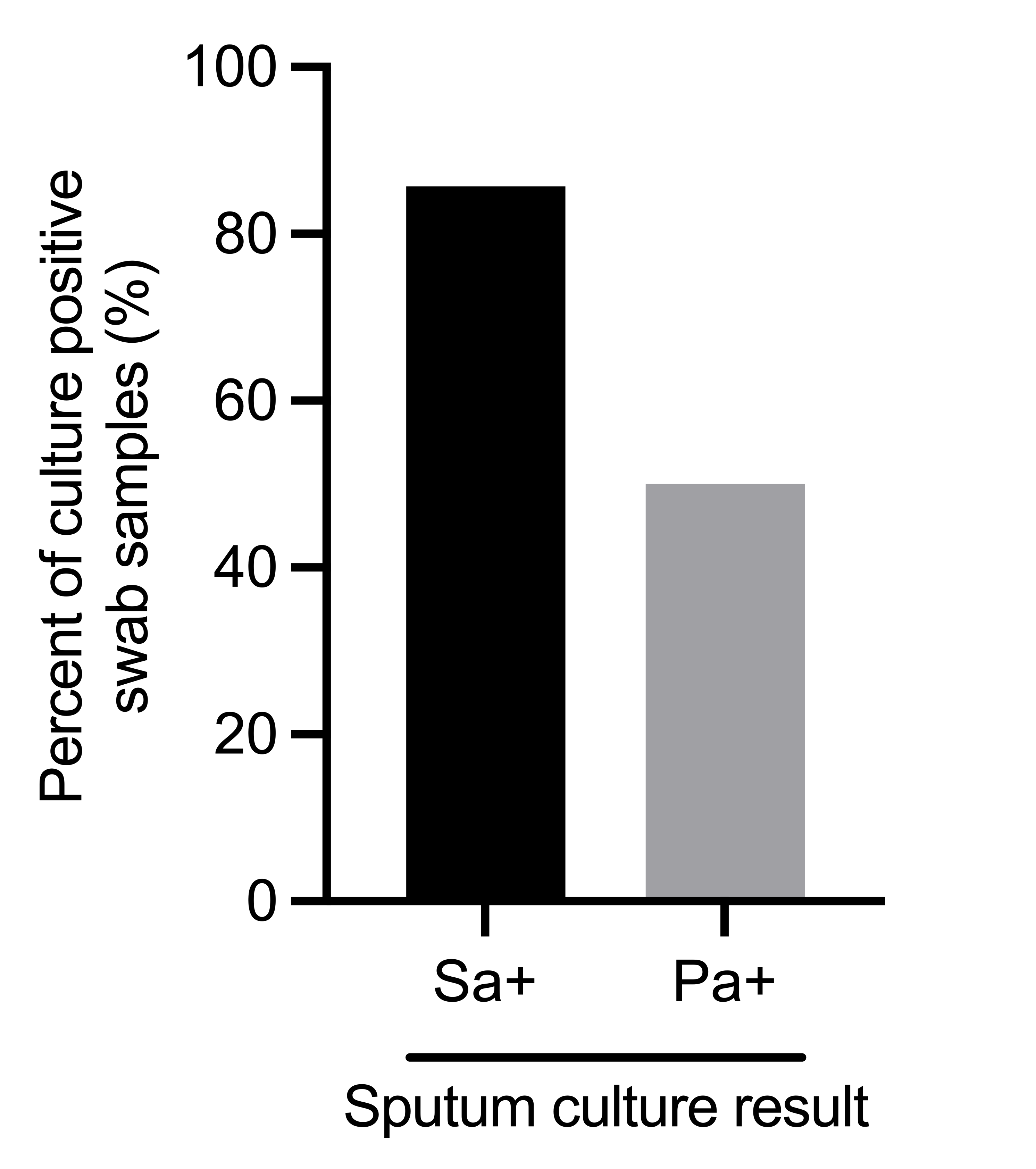

Supplement: FIG S5 [file mbio.03148-21-sf005.tif]

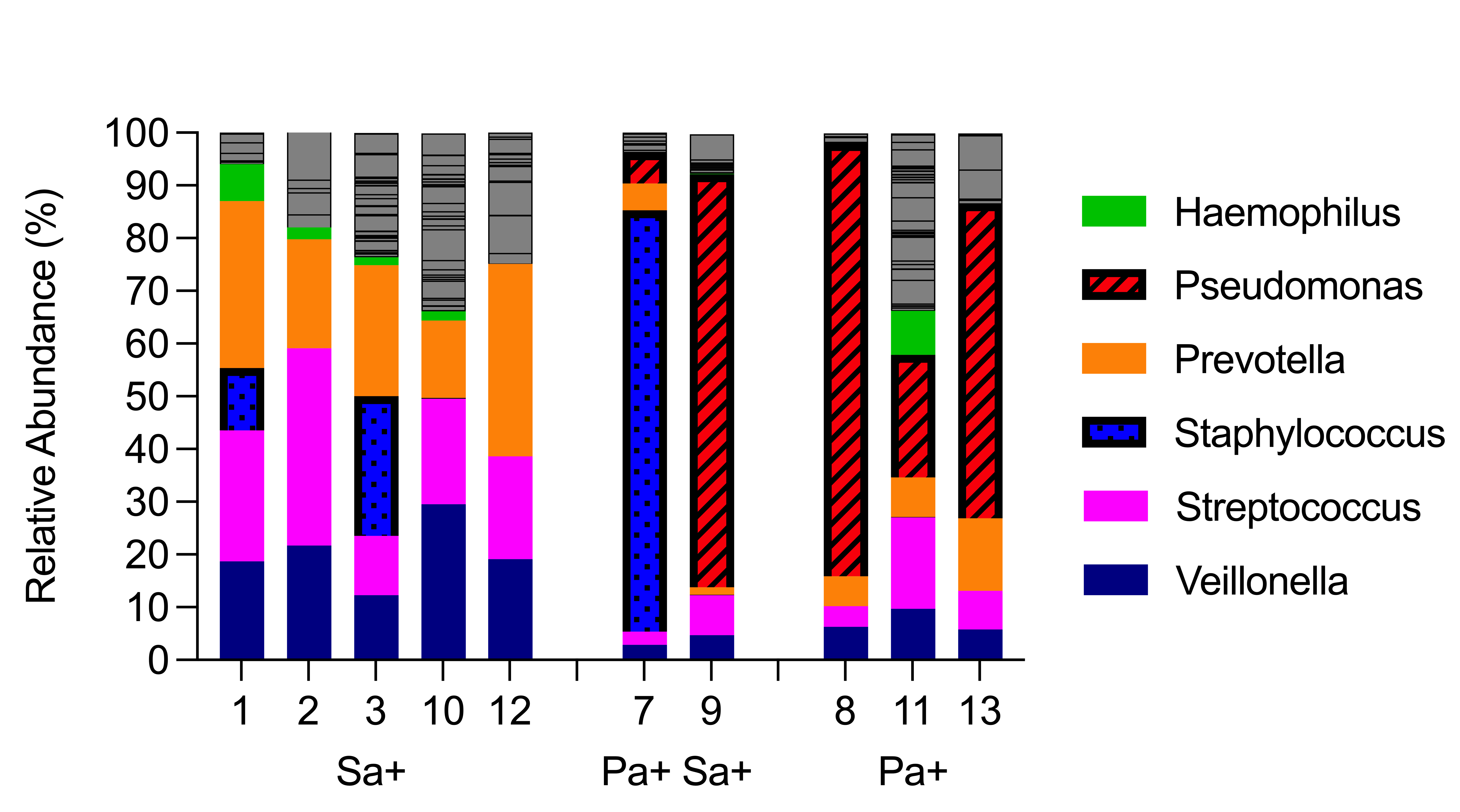

Supplement: FIG S6 [file mbio.03148-21-sf006.tif]
