# Supplementary material for: Combining Ivacaftor and Intensive Antibiotics Achieves Limited Clearance of Cystic Fibrosis Infections
Source: mBio. 2021 Dec 14;12(6):e03148-21. doi: 10.1128/mbio.03148-21 (PMC8669489; doi:10.1128/mbio.03148-21)
Supplement: TABLE S3 [file mbio.03148-21-st003.docx]

**Table S3.** Infection histories of Subjects 3 and 9.

Culture was performed by the clinical diagnostic lab.
